# A Polysaccharide-Rich Ingredient from *Hypericum perforatum* L. Ameliorates Depression-like and Post-Traumatic Stress Disorder-like Symptoms in Mouse Models

**DOI:** 10.3390/nu17203222

**Published:** 2025-10-14

**Authors:** Zi-Jia Jin, Shuai-Ming Zhu, Fu-Yao Luo, Yue Sun, Chun-Xue Gao, Ting Feng, Hao Ma, Rui Xue, Chang-Wei Li, Lei An, You-Zhi Zhang

**Affiliations:** 1 Key Laboratory of Geriatric Nutrition and Health, Ministry of Education, Beijing Technology and Business University, Beijing 100048, China; 2 State Key Laboratory of National Security Specially Needed Medicines, Academy of Military Medical Sciences, Beijing 100850, China; 3 School of Pharmacy, North China University of Science and Technology, Tangshan 063210, China; 4 Nanjing University of Chinese Medicine, Nanjing 210046, China

**Keywords:** *Hypericum perforatum* L., polysaccharide, antidepressant, anti-PTSD, gut microbiota

## Abstract

**Background/Objectives**: *Hypericum perforatum* L. (*H. perforatum*), commonly known as St. John’s wort, has been widely used in clinical practice to treat mental disorders. Previous studies and clinical applications have primarily focused on its alcohol-soluble ingredients. Our research was designed to investigate the physicochemical properties, antidepressant-like effects, and anti-post-traumatic stress disorder (PTSD)-like effects of the alcohol-insoluble polysaccharide-rich ingredients from *H. perforatum*. Meanwhile, the underlying mechanisms were elucidated. **Methods**: The physicochemical properties of two polysaccharide-rich ingredients, designated as HPP1 and HPP2, were characterized using colorimetric assay, capillary electrophoresis, high-performance gel permeation chromatography, and fourier transform infrared spectroscopy. Behavioral despair tests were conducted to rapidly assess and compare their antidepressant-like effects in mice. Subsequently, behavioral despair mice and foot-shock mice were established to thoroughly explore the impact of HPP2 on depression-like and PTSD-like symptoms. The effects of HPP2 on cerebral pathological changes, neurotrophic factors, and gut microbiota in foot-shock mice were detected through hematoxylin & eosin staining, immunofluorescence staining, and 16S rDNA (V3 + V4 regions) gene sequencing. **Results**: HPP1 and HPP2 are predominantly composed of arabinose, glucose, galactose, mannose, and galacturonic acid. The molecular weight distribution of HPP1 ranges from 1133 to 67,278 Da, whereas that of HPP2 extends from 1493 to 38,407 Da. Acute pre-treatment with HPP1 or HPP2 (200 mg/kg, i.g.) could reduce mice’s immobility in behavioral despair tests, with HPP2 exhibiting superior efficacy. Additionally, both acute and sub-chronic pre-treatment with HPP2 (50, 200, and 800 mg/kg, i.g.) effectively alleviated depression-like symptoms in behavioral despair mice. Prolonged pre-treatment with HPP2 (200 mg/kg, i.g.) also mitigated the slow increase in body weight and behavioral abnormalities in foot-shock mice. Furthermore, HPP2 (200 mg/kg) successfully restored hippocampal histomorphological abnormalities, neurotrophic disturbance, and dysregulation of the gut microbiota in foot-shock mice. **Conclusions**: HPP2 exerts noteworthy antidepressant-like and anti-PTSD-like impact in mouse models via multiple targets, indicating a potential therapeutic candidate in depression and PTSD therapy.

## 1. Introduction

Depression and post-traumatic stress disorder (PTSD) rank among the most common and disabling psychiatric illnesses globally, impacting millions each year, significantly impairing quality of life and imposing substantial economic burdens. Depression is characterised by persistent low mood and/or anhedonia for at least 2 weeks accompanied by cognitive, vegetative and metabolic symptoms. PTSD usually develops after exposure to death or serious injury and manifests with intrusive re-experiencing, avoidance, negative alterations in cognition/mood, and hyper-arousal [1,2]. Despite the availability of various synthetic medicines, many patients still encounter issues such as limited efficacy, adverse effects, or treatment resistance [3]. Even more troubling, the Food and Drug Administration has approved only two first-line agents for PTSD, sertraline hydrochloride (SER) and paroxetine hydrochloride, underscoring the pressing demand for innovative treatment strategies [4]. In recent years, the potential therapeutic benefits of natural ingredients, particularly polysaccharides derived from medicinal plants, have garnered increasing attention. These polysaccharides have demonstrated antidepressant-like and anti-PTSD-like effects in preclinical studies through various mechanisms, including modulation of neurotransmitter systems, reduction in oxidative stress and neuroinflammation, and regulation of the gut–brain axis [5,6,7,8].

*Hypericum perforatum* L. (*H. perforatum*), widely recognized as St. John’s wort, has long been used as a prominent complementary and alternative plant for its antidepressant properties, particularly in China and German-speaking countries [9,10]. Antidepressant medicines such as *Luyoutai* (LYT) and *Shuganjieyu* capsule (SG), which are mainly composed of alcohol-soluble extracts from *H. perforatum*, are widely used as first-line monotherapies for treating mild to moderate depression [11,12]. Chemical studies have pinpointed several alcohol-soluble ingredients, including phloroglucines, naphthodianthrones, and flavonoids, which are attributed to the antidepressant effects of *H. perforatum* [13]. These alcohol-soluble constituents are believed to act by blocking the synaptic uptake of serotonin (5-HT), noradrenaline (NE), and dopamine, tuning glutamatergic and gamma-aminobutyric acid circuit, and enhancing neuroplasticity [14]. Commercial extracts are typically standardized based on their hypericin or hyperforin content, both of which have demonstrated antidepressant-like activity [15]. However, the modulatory effects of alcohol-insoluble polysaccharides, a major class of ingredients from *H. perforatum*, on mental disorders have not been evaluated in typical animal models. This gap in knowledge highlights the need for further research to explore the potential of polysaccharides from *H. perforatum* (HPP) for managing depressive and PTSD symptoms.

In the study, two novel polysaccharide-rich ingredients, termed HPP1 and HPP2, were prepared from *H. perforatum* using distinct extraction methods. Their contents, monosaccharide compositions, molecular weight (MW) range, and functional motifs were analysed through colorimetric assay, capillary electrophoresis (CE), high-performance gel permeation chromatography (HPGPC), and fourier transform infrared (FT-IR) spectroscopy [3]. Forced swimming test (FST) and tail suspension test (TST) were first conducted to promptly compare the antidepressant-like effects of HPP1 and HPP2 at a dose of 200 mg/kg in mice [16]. Then despair-model mice were next generated to examine the antidepressant-like impact of acute and sub-chronic HPP2 pre-treatment (50, 200, 800 mg/kg, i.g.). Subsequently, the foot-shock mice, a typical PTSD-like animal model, were developed based on prior studies to elucidate the anti-PTSD-like effects of sub-chronic pre-treatment with HPP2 (200 mg/kg) [17]. Furthermore, the effects of HPP2 on pathological changes and neurotrophic factors in the hippocampus, as well as on the gut microbiota, were investigated using hematoxylin & eosin (HE) staining, immunofluorescence (IF) staining, and 16S rDNA (V3 + V4 regions) gene sequencing in foot-shock mice to elucidate the underlying mechanisms [3,18,19].

## 2. Materials and Methods

### 2.1. Extractions and Physicochemical Characteristics of HPP1 and HPP2

#### 2.1.1. Plant Material

Air-dried *H. perforatum* aerial parts (batch 20220914) were sourced from Beijing Tongrentang (Beijing, China), grown in Guangxi, and processed by Beijing Bencao Fangyuan (Beijing, China).

#### 2.1.2. Extraction

Dried material (200 g) was boiled three times in 2 L of distilled water (1 h each). The pooled filtrate was centrifuged, concentrated to 200 mL, mixed with 3 vol 95% ethanol, and kept at 4 °C overnight. The precipitate collected by centrifugation (3000 rpm, 10 min) was redissolved, concentrated at 60 °C, and lyophilised to give the polysaccharide-rich ingredient designated as HPP1.

Another 200 g batch was first extracted twice with 70% ethanol (2 L, 1 h each). The residue was then boiled three times in water (2 L, 1 h), the pooled aqueous filtrate centrifuged, concentrated to 200 mL, and precipitated with 3 vol 95% ethanol at 4 °C overnight. The pellet collected by centrifugation (3000 rpm, 10 min) was redissolved, concentrated at 60 °C, and lyophilised to afford the polysaccharide-rich ingredient HPP2.

#### 2.1.3. Physicochemical Characteristic

The quantities of total neutral sugars and uronic acids in HPP1 and HPP2 were measured via colorimetric assays. CE was utilized to examine monosaccharide compositions. The MW distribution was evaluated using HPGPC on a Waters Delta 600 system (Waters Corporation, Milford, CT, USA). FT-IR analysis mapped the chemical motifs of HPP1 and HPP2, with spectra captured within the 400–4000 cm^−1^ range. Further details on the instruments and standards employed are available in our prior study. Detailed information regarding the instruments and standards utilized can be found in our previous research [3].

### 2.2. Comparison of Antidepressant-like Effects of Acute Pre-Treatment with HPP1 and HPP2 in Mice

#### 2.2.1. Animals and Grouping

Eighty male ICR mice (20 ± 2 g, SPF Biological Technology, Beijing, China) were housed at 24 ± 1 °C on a 12 h light-dark cycle with ad libitum food and water. After 7 days acclimation, animals were randomly assigned to four groups (n = 20): the control group (Vehicle; 20 mL/kg, i.g.), the duloxetine hydrochloride (DLX)_20 group (DLX; 20 mg/kg, i.g.), the HPP1_200 group (HPP1; 200 mg/kg, i.g.), and the HPP2_200 group (HPP2; 200 mg/kg, i.g.). All procedures followed the Institutional Animal Care and Use Committee guidelines (approval IACUC-DWZX-2021-618) and every effort was made to minimise suffering. Notably, the ICR mice were chosen because their heterogeneous genetic background more closely mimic the variability encountered in human depression, thereby increasing translational validity of pharmacological interventions.

#### 2.2.2. Drug Treatment

Following 7 days of acclimation, mice received oral doses of DLX (1304002; Shanghai Wandai Pharmaceutical, Shanghai, China), HPP1, or HPP2. Dose levels were extrapolated from published data, pilot studies and human-equivalent scaling [16]. Controls received the same volume of vehicle.

#### 2.2.3. Behavioral Testing

FST and TST are most commonly used for studying depression and rapdily predicting the efficacy of antidepressant drugs in rodents, in which animals are placed in a stressful situation and then exhibit behavioral despair defined as immobility time in response to the inescapable stressor. One hour post-dose, ten mice per group were subjected to the FST and another ten to the TST to evaluate despair-like responses to inescapable stress. The procedures for the FST and TST were carried out as described in our prior publication [3]. FST quantified despair by timing the last 4 min of a 6 min forced-swim in 25 °C water (15 cm depth, cylinder 20 × 10 cm); immobility (passive floating) was scored by an investigator blinded to treatment. Mice undergoing the TST were taped 1 cm from the tail tip and hung 15 cm above the floor for 6 min; immobility during the final 4 min was scored by a blinded observer.

### 2.3. Antidepressant-like Effects of Acute Pre-Treatment with HPP2 in Mice

#### 2.3.1. Animals and Grouping

One hundred male ICR mice (20 ± 2 g) from a single supplier were randomly assigned to five groups (n = 20): the control group (Vehicle; 20 mL/kg, i.g.), the SG_200 group (SG; 200 mg/kg, i.g.), the HPP2_50 group (HPP2; 50 mg/kg, i.g.), the HPP2_200 group (HPP2; 200 mg/kg, i.g.), and the HPP2_800 group (HPP2; 800 mg/kg, i.g.). Housing and care followed the ethical procedures described above.

#### 2.3.2. Drug Treatment

Following a 7-day acclimation period to the environment, the mice were administered SG (210316; Sichuan Jishengtang, Chengdu, China) and HPP2 at the previously specified dosages via oral gavage. The dosage of SG was determined based on our earlier study [3]. Controls received the same volume of vehicle.

#### 2.3.3. Behavioral Testing

Despair-like behaviour was evaluated with the FST and TST after a single dose of HPP2 pre-treatment. Both tests followed our published protocols.

### 2.4. Antidepressant-like Effects of Sub-Chronic Pre-Treatment with HPP2 in Mice

#### 2.4.1. Animals and Grouping

Fifty male ICR mice (20 ± 2 g) from the same supplier were randomly assigned to five groups (n = 10): the control group (Vehicle; 20 mL/kg, i.g.), the LYT_150 group (LYT; 150 mg/kg, i.g.), the HPP2_50 group (HPP2; 50 mg/kg, i.g.), the HPP2_200 group (HPP2; 200 mg/kg, i.g.), and the HPP2_800 group (HPP2; 800 mg/kg, i.g.). Housing and care followed the ethical procedures described above.

#### 2.4.2. Drug Treatment

After environmental adaptation for 7 days, the oral administration of LYT (0460522), procured from Dr. Willmar Schwabe GmbH & Co. KG. (Karlsruhe, Germany), and HPP2 at the aforementioned doses was conducted. The dosage of LYT was determined via body surface area-based dose conversion from humans to mice. Controls received the same volume of vehicle.

#### 2.4.3. Behavioral Testing

On days 7–9 after daily dosing, mice underwent TST, FST, and reserpine-induced ptosis and hypothermia test, using the same published protocols [16]. In the reserpine-induced ptosis and hypothermia test, 60 min post-drug, 2.5 mg/kg reserpine (0.2% acetic acid, i.p.) was administered; ptosis was scored 90 min later on a 0–4 scale (0 = eyes open, 1 = one-quarter closed, 2 = half closed, 3 = three-quarters closed, 4 = completely closed). Rectal temperature was recorded at 120 min.

### 2.5. Anti-PTSD-like Effects of Sub-Chronic Pre-Treatment with HPP2 in Mice

#### 2.5.1. Animals and Grouping

An additional thirty-six male ICR mice (20 ± 2 g) from the same supplier were randomly assigned to four groups (n = 9): the control group (Vehicle + Sham; 20 mL/kg, i.g.), the model group (Vehicle + Foot-shock; 20 mL/kg, i.g.), the SER_15 group (SER + Foot-shock; 15 mg/kg, i.g.), and the HPP2_200 group (HPP2 + Foot-shock; 200 mg/kg, i.g.). Housing and care followed the ethical procedures described above.

#### 2.5.2. Drug Treatment

After acclimation, mice received SER (S838258; Shanghai Macklin Biochemical, Shanghai, China) and HPP2 at the stated dose by daily gavage for 10 days. The dosage of SER was determined in accordance with our prior research [20]. The control and model groups received equivolume vehicle orally.

#### 2.5.3. Foot-Shock Procedure

On day 7, foot-shocks (Intensity: 0.8 mA; Duration: 2 s; Interval: 120 s) were delivered through a grid floor connected to an isolated generator (Shanghai Jiliang Software Technology, Shanghai, China). After a 120 s acclimation, each mouse received five inescapable shocks over 10 min. Controls remained in the chamber for the same period without shocks.

#### 2.5.4. Behavioral Testing

On day 8, the contextual freezing test (CFT) was assessed by returning each mouse to the shock chamber for 2 min without shocks. Freezing, absence of movement except breathing, was recorded automatically (Shanghai Jiliang Software Technology, Shanghai, China) and expressed as total freezing time (s) [20].

On day 9, anxiety was assessed in the elevated plus maze test (EPMT): two open (50 × 10 cm) and two closed arms (50 × 10 cm, 14 cm walls) joined by a 10 × 10 cm central platform 50 cm above floor. Mice were placed head-facing an open arm. Time and distance in open arms during 5 min were tracked with Dig-Behv software V2.0 (Shanghai Xinruan Information Technology, Shanghai, China) [21].

The open field test (OFT) was also conducted to assess the anxiety degree of mice on day 10. Briefly, all animals were placed in an activity test chamber (50 cm × 50 cm × 50 cm) using Dig-Behv video tracking system software (Nanjing Calvin Biotechnology, Nanjing, China). Total distance (cm) and percentage of centre distance were logged for 5 min. Percentage of centre distance (%) = (Centre distance/Total distance) × 100%.

#### 2.5.5. Sample Collection

After behavioural tests, three foot-shock mice per group were anaesthetised with isoflurane, perfused with 4% paraformaldehyde and processed for HE/IF staining. Colonic contents from the remaining animals were collected for microbiota analysis.

### 2.6. HE Staining

Brains from perfused, isoflurane-anaesthetised mice were cut into 5 µm sections, processed (deparaffinised, rehydrated, stained, dehydrated, cleared and mounted) and imaged by light microscopy. Intact neurons appeared round with distinct nuclei. Deeply stained, pyknotic nuclei lacking clear cytoplasmic borders were counted as degenerate using ImageJ 1.41 (National Institutes of Health) in the hippocampal dentate gyrus (DG) and cornu ammonis (CA) regions [21].

### 2.7. IF Staining

Some slices from Section 2.6 were blocked (0.3% Triton X-100 + 1% BSA, 60 min), incubated with an antibody specific to brain-derived neurotrophic factor (BDNF) (4 °C, overnight), then with Cy3-conjugated goat anti-rabbit IgG (room temp, 2 h, dark) and 4′,6-diamidino-2-phenylindole (DAPI) for 10 min. Fluorescence was captured on a Dragonfly 200 confocal (Andor) and analysed in CaseViewer 2.4.0. BDNF signal was quantified in DG, CA1 and CA2/CA3 [22]. Antibodies: BDNF (AB108319, Abcam, Cambridge, United Kingdom), Cy3-IgG (AP136C, Sigma, San Francisco, CA, USA), DAPI (D9542, Sigma, San Francisco, CA, USA).

### 2.8. Gut Microbiota Analysis

Faecal DNA was isolated with the E.Z.N.A.^®^ Stool kit, the 16S V3–V4 region amplified and sequenced on the Illumina MiSeq platform. After quality filtering and merging, reads were resolved into amplicon sequence variants (ASVs) and analysed in QIIME 2. α-diversity (chao, shannon, ace, and sobs) and β-diversity (principal coordinate analysis, PCoA) were calculated, and taxonomic profiles generated on phylum and genus levels across different groups [3].

### 2.9. Statistical Analysis

Behavioural and biochemical data presented as mean ± standard error media (SEM) were analysed in SPSS 16.0 and plotted with GraphPad Prism 6.01. Two-group comparisons used Student’s *t*-test; multi-group comparisons used one-way analysis of variance (ANOVA) followed by LSD. *p* < 0.05 was considered significant and 0.05 ≤ *p* < 0.1 was noted as a trend. Gut-microbiota statistics (mean ± SEM) were run on the Majorbio cloud platform (Shanghai, China) with Student’s *t*-test for inter-group comparisons. All plots were generated online.

## 3. Results

### 3.1. Extractions and Physicochemical Characteristics of HPP1 and HPP2

**Figure 1:**
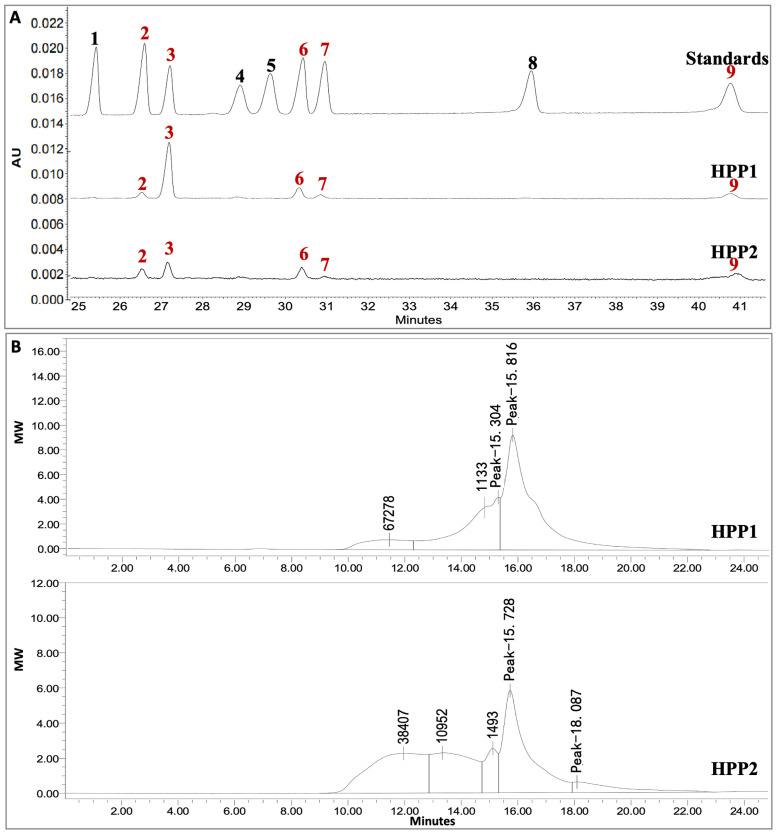
Monosaccharide composition and MW distribution of HPP1 and HPP2. (**A**) CE of 1-phenyl-3-methyl-5-pyrazolone-labeled standards and monosaccharides (245 nm), 1: xylose, 2: arabinose, 3: glucose, 4: rhamnose, 5: fucose, 6: galactose, 7: mannose, 8: glucuronic acid, 9: galacturonic acid. (**B**) HPGPC on TSK-gel G-3000swxl column detected by the refractive index detector.

HPP1 and HPP2 were isolated in 5.1% and 2.4% yield, respectively. As shown in Table 1, HPP1 mainly contains 44.3% neutral sugar plus 58.6% uronic acid, while HPP2 contains 35.6% neutral sugar plus 57.4% uronic acid. Both ingredients are heteropolysaccharides of arabinose, glucose, galactose, mannose, and galacturonic acid (HPP1 molar ratio 1.00:9.03:2.44:0.75:2.32; HPP2 1.00:1.42:1.41:0.19:0.57) (Table 1, Figure 1A). HPGPC on TSK-gel G-3000swxl column gave MW ranges of 1133 to 67278 Da for HPP1 and 1493 to 38407 Da for HPP2 (Figure 1B).

**Figure 2:**
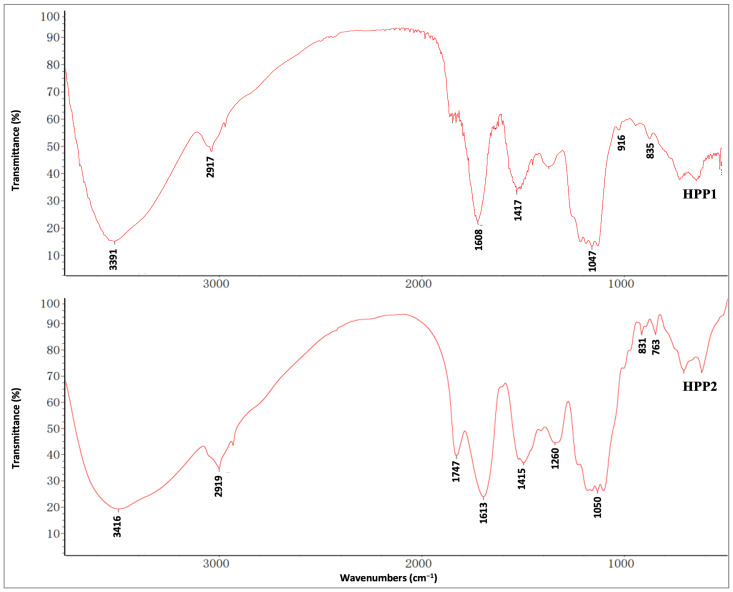
FT-IR spectra of HPP1 and HPP2 recorded by the KBr disk method.

FT-IR spectra (Figure 2) reveal the key functional signatures of HPP1 and HPP2: broad bands at 3391/3416 and 1417/1415 cm^−1^ trace O–H stretch/bend, 2917/2919 cm^−1^ reflects sugar C–H stretch, 1608/1613 cm^−1^ maps C=O/carboxyl stretch, and 1047/1050 cm^−1^ arises from C–O–C pyranose breathing; anomeric peaks at 835/916 cm^−1^ (α/β) for HPP1 and 831/761 cm^−1^ (α) for HPP2 confirm glycosidic linkage patterns [23].

### 3.2. Comparison of Effects of Acute Pre-Treatment with HPP1 and HPP2 in Behavioral Despair Mice

**Figure 3:**
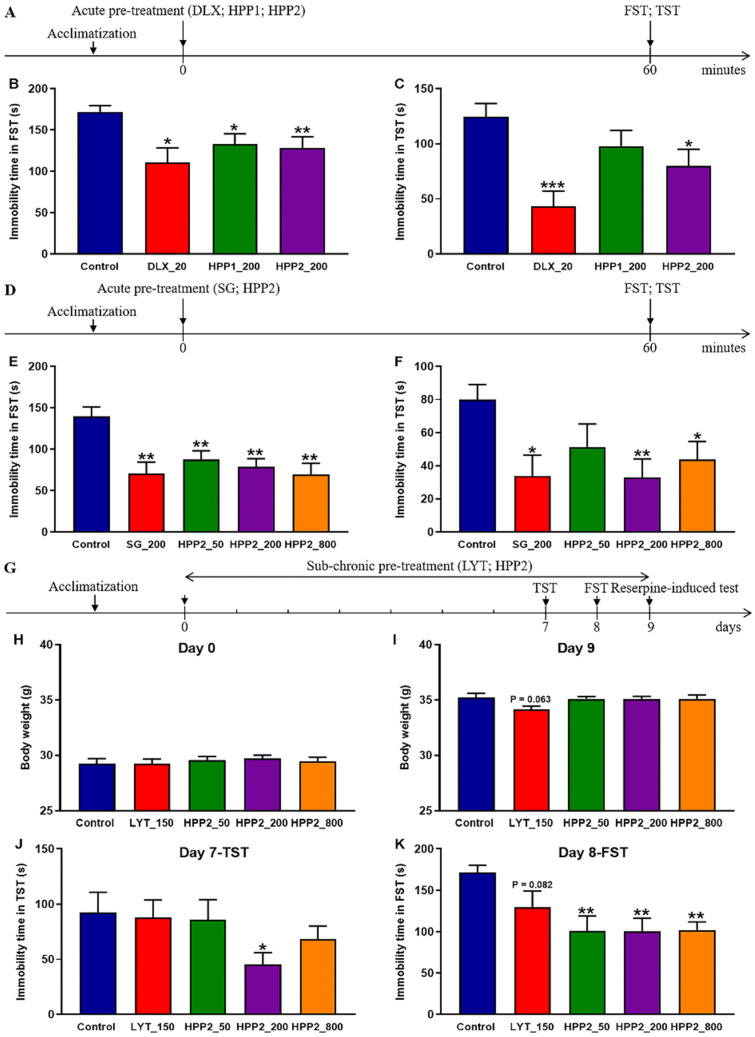
Antidepressant-like effects of HPP1 and HPP2 in behavioral despair mice. (**A**,**D**,**G**) Schematic illustrations of experiments. (**B**,**C**) Effects of acute pre-treatment with HPP1 and HPP2 in FST and TST. (**E**,**F**) Effects of acute pre-treatment with HPP2 in FST and TST. (**H**–**K**) Effects of sub-chronic pre-treatment with HPP2 in body weight, TST and FST. Measurement data were analyzed by Student’s *t*-test or one-way ANOVA (post hoc LSD test), and presented as mean ± SEM (n = 10). * *p* < 0.05, ** *p* < 0.01, *** *p* < 0.001 relative to the control group.

As shown in Figure 3A, FST and TST were first used for a rapid comparison of the antidepressant-like potential of the two polysaccharide-rich ingredients. Figure 3B,C demonstrated that acute DLX pre-treatment (20 mg/kg) reduced immobility in both tests (*p* < 0.05 and *p* < 0.001, respectively), confirming the validity of the despair-based model. At 200 mg/kg, HPP1 shortened FST immobility (*p* < 0.05), whereas the same dose of HPP2 was active in both FST (*p* < 0.01) and TST (*p* < 0.05), indicating a stronger stress-buffering effect for HPP2 under the present conditions.

### 3.3. Effects of Acute Pre-Treatment with HPP2 in Behavioral Despair Mice

Next, a comprehensive evaluation of antidepressant-like effects of HPP2 was profiled comprehensively in the behavioural despair paradigm (Figure 3D). Acute SG pre-treatment (200 mg/kg) lowered immobility in both FST and TST versus vehicle (*p* < 0.01, *p* < 0.05; Figure 3E,F). Further post hoc tests showed HPP2 (50, 200, 800 mg/kg) equally effective in the FST (all *p* < 0.01), while in the TST only the 200 and 800 mg/kg doses reached significance (*p* < 0.01, *p* < 0.05, respectively). The 50 mg/kg dose shortened immobility but did not achieve significance (*p* > 0.1).

### 3.4. Effects of Sub-Chronic Pre-Treatment with HPP2 in Behavioral Despair Mice

As depicted in Figure 3G, a 9-day prolonged pre-treatment regimen, complemented by behavioral assessments on days 7, 8, and 9, was implemented to appraise the antidepressant-like effects of sub-chronic HPP2 pre-treatment in mice. The data presented in Figure 3H demonstrated that no significant variances in body weight were discerned prior to the commencement of drug pre-treatment. However, by day 9, mice in the LYT_150 group exhibited a gradual decline in body weight relative to the control group, albeit this decrement did not attain statistical significance (Figure 3I; *p* = 0.063).

On day 7, the TST unveiled that HPP2 at a dose of 200 mg/kg significantly reduced the immobility time in mice (Figure 3J; *p* < 0.05), while no significant differences were detected between the LYT_150, HPP2_50, or HPP2_800 groups and the control group (*p* > 0.1). On day 8, post hoc analysis indicated that HPP2 at 50, 200, and 800 mg/kg doses produced notable effects on the immobility time in the FST (*p* < 0.01), and the LYT_150 group also exhibited a gradual decrease compared to the control group, although this decrease did not reach statistical significance (Figure 3K; *p* = 0.082).

On day 9, the reserpine challenge was used to probe 5-HT/NE function in mice. Table 2 showed that 2.5 mg/kg reserpine elicited pronounced ptosis and hypothermia. Nine-day pre-treatment with LYT (150 mg/kg) or HPP2 (200 and 800 mg/kg) significantly reduced ptosis severity (*p* < 0.05 or *p* < 0.001). LYT also lessened hypothermia, but this change did not attain significance (*p* > 0.1).

### 3.5. Effects of Sub-Chronic Pre-Treatment with HPP2 in Foot-Shock Mice

**Figure 4:**
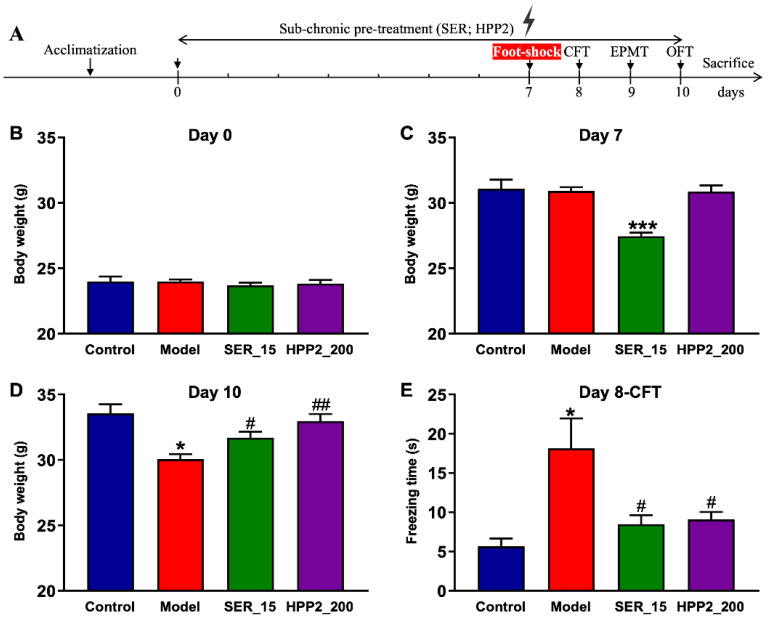
Anti-PTSD-like effects of HPP2 on body weight and traumatic re-experiencing in foot-shock mice. (**A**) Schematic illustration of experiments. (**B**–**D**) Effects of sub-chronic pre-treatment with HPP2 in body weight. (**E**) Effects of sub-chronic pre-treatment with HPP2 in CFT. Measurement data were analyzed by Student’s *t*-test and represented as mean ± SEM (n = 9). * *p* < 0.05, *** *p* < 0.001 relative to the control group; # *p* < 0.05, ## *p* < 0.01 in contrast to the model group.

Figure 4A outlines the foot-shock protocol used to probe sub-chronic HPP2 pre-treatment for anti-PTSD-like activity. Baseline body weight did not differ among groups (Figure 4B). However, mice in the SER_15 group weighed less than controls on day 7 (Figure 4C), and shocked animals showed a marked drop by day 10 (*p* < 0.05). Both SER (15 mg/kg^1^) and HPP2 (200 mg/kg) attenuated this shock-induced growth retardation (Figure 4D; *p* < 0.05, *p* < 0.01, respectively).

To explore the impact of drug pre-treatment on traumatic re-experiencing, a core symptom of PTSD, the CFT was initially conducted on day 8. As depicted in Figure 4E, the model mice demonstrated a significantly longer freezing time (*p* < 0.05), indicating that the PTSD-like model with a fear response to the context associated with traumatic events was successfully established in our study. Concurrently, SER pre-treatment (15 mg/kg) for 8 days substantially reduced freezing time relative to the control mice (*p* < 0.05). HPP2 (200 mg/kg) was also observed to ameliorate the core symptom of PTSD-like mice (*p* < 0.05). Together, these results confirm the model’s face validity and highlight HPP2’s promise for curbing traumatic re-experiencing.

**Figure 5:**
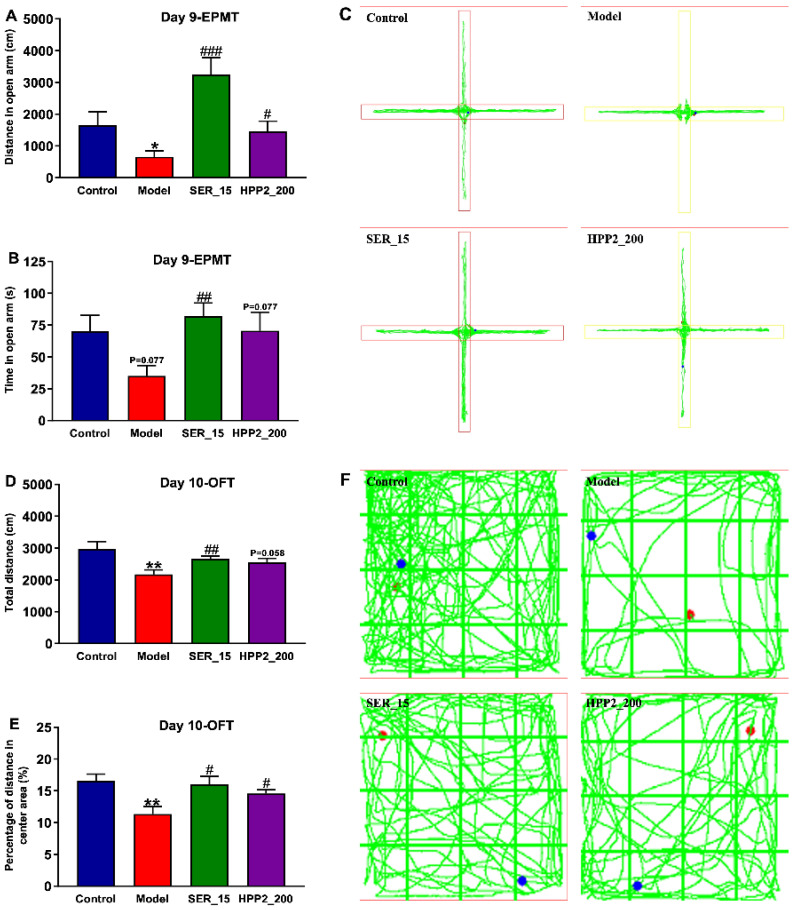
Anti-PTSD-like effects of HPP2 on increased vigilance in foot-shock mice. (**A**–**C**) Effects of sub-chronic pre-treatment with HPP2 in EPMT. (**D**–**F**) Effects of sub-chronic pre-treatment with HPP2 in OFT. Measurement data were analyzed by Student’s *t*-test, and represented as mean ± SEM (n = 9). * *p* < 0.05, ** *p* < 0.01 relative to the control group; # *p* < 0.05, ## *p* < 0.01, ### *p* < 0.001 in contrast to the model group. In (**F**), blue dot indicates the starting point, and red dot indicates the ending point.

Increased vigilance, another PTSD hallmark, was assessed with EPMT (day 9) and OFT (day 10). In EPMT, the model mice entered the open arm less often and covered less distance there (Figure 5A–C; *p* < 0.05, *p* = 0.077). In OFT, they travelled less and spent a lower percentage of distance in the centre (Figure 5D–F; *p* < 0.01), confirming abnormal activity and anxiety-like behaviors. SER (15 mg/kg) reversed these deficits (EPMT: *p* < 0.001, *p* < 0.01; OFT: *p* < 0.01, *p* < 0.05); HPP2 (200 mg/kg) also effectively normalized the aforementioned indicators (*p* < 0.05, *p* = 0.058, or *p* = 0.077). These findings demonstrated their therapeutic effects on the anxiety-like behaviors of model mice.

### 3.6. Effects of Sub-Chronic Pre-Treatment with HPP2 on Hippocampal Histomorphological Abnormality in Foot-Shock Mice

**Figure 6:**
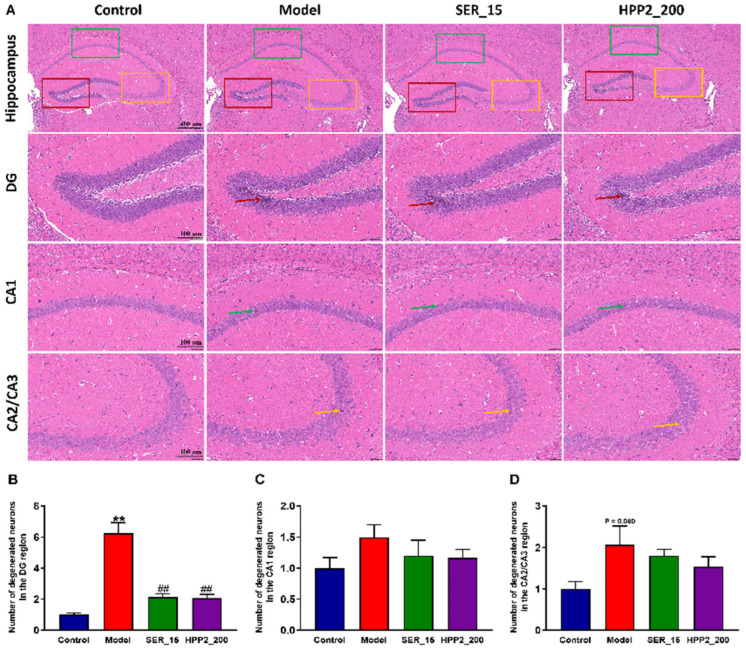
Effects of sub-chronic pre-treatment with HPP2 on hippocampal histomorphological abnormality in foot-shock mice. (**A**) Representative HE staining images in the hippocampal region (scale bar = 400 or 100 μm). In the model group, marked nuclear condensation within the DG and a disorganized, loosely arranged neuronal structure in the CA2/CA3 regions were observed. These abnormalities were attenuated by SER or HPP2 pre-treatment. (**B**–**D**) Quantification of degenerated neurons in the hippocampal region. “1” represents 100% of the signal relative to the control group. Measurement data were analyzed by Student’s *t*-test and represented as mean ± SEM (n = 3). ** *p* < 0.01 relative to the control group; ## *p* < 0.01 in contrast to the model group.

Subsequent to the completion of behavioral assessments, the brains of the mice were extracted, and histopathological alterations within the hippocampus were scrutinized via HE staining. Notably, in the foot-shock group, there was a pronounced augmentation in nuclear condensation within the DG region, juxtaposed with a disorganized and loosely structured neuronal layout in the CA2/CA3 region (Figure 6A). Within the brain samples from the model group, extensive neuronal necrosis and profound nuclear condensation staining were evident in the hippocampal DG area (Figure 6B; *p* < 0.01). When juxtaposed with the model group, the hippocampal tissues in the SER_15 and HPP2_200 groups exhibited a more normalized appearance. This normalization was characterized by a higher neuronal count and a more orderly, regular structure in the hippocampal DG area, accompanied by well-defined cell morphology and distinct nuclei (*p* < 0.01). Additionally, there was a moderate increase in the number of degenerated neurons in the CA1 and CA2/CA3 regions of the model mice. While statistical significance was not achieved, both SER (15 mg/kg) and HPP2 (200 mg/kg) demonstrated the capacity to partially mitigate the previously mentioned histomorphological abnormalities (Figure 6C,D; *p* > 0.1).

### 3.7. Effects of Sub-Chronic Pre-Treatment with HPP2 on Hippocampal BDNF Expression in Foot-Shock Mice

**Figure 7:**
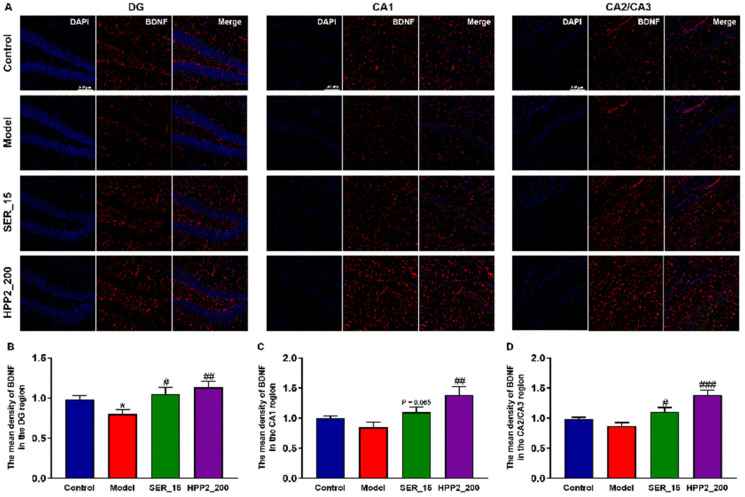
Effects of sub-chronic pre-treatment with HPP2 on hippocampal BDNF expression in foot-shock mice. (**A**) Representative IF staining images in the hippocampal region (scale bar = 100 μm). (**B**–**D**) Quantification of BDNF expression in the hippocampal region. “1” represents 100% of the signal relative to the control group. Measurement data were analyzed by Student’s *t*-test and represented as mean ± SEM (n = 3). * *p* < 0.05 relative to the control group; # *p* < 0.05, ## *p* < 0.01, ### *p* < 0.001 in contrast to the model group.

BDNF, a key neurotrophin, drives neuronal survival and plasticity. To explore the impact of sub-chronic pre-treatment with HPP2 on neurotrophic disturbance, we employed the IF assay to quantify BDNF levels in the hippocampi of foot-shock mice. As shown in Figure 7A,B, the foot-shock procedure lowered BDNF level in DG (*p* < 0.01). BDNF expression in the hippocampal CA1 and CA2/CA3 regions was similarly reduced to some extent (Figure 7C,D; *p* > 0.1). Concurrently, nine-day HPP2 pre-treatment (200 mg/kg) restored BDNF to control levels throughout the hippocampus (*p* < 0.001 or *p* < 0.01), whereas SER (15 mg/kg) yielded partial recovery (*p* < 0.05, *p* = 0.058, and *p* < 0.05, respectively).

### 3.8. Effects of Sub-Chronic Pre-Treatment with HPP2 on Gut Microbiota Dysregulation in Foot-Shock Mice

**Figure 8:**
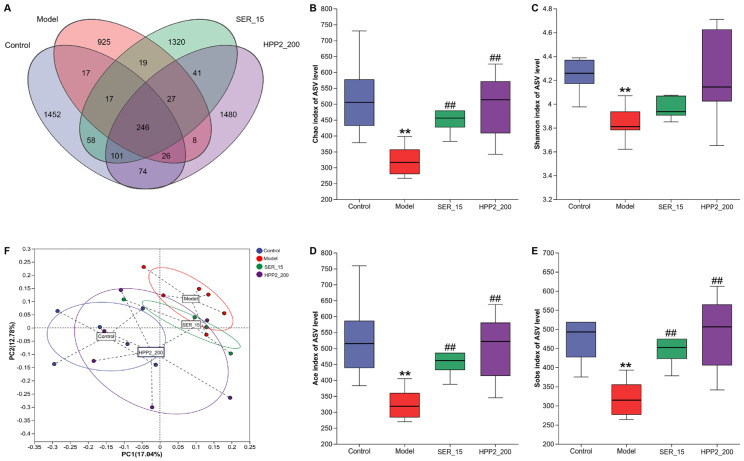
Effects of sub-chronic pre-treatment with HPP2 on the richness and similarity degree of gut microbiota among microbial communities in foot-shock mice. (**A**) Venn disgram on ASV level among different groups. (**B**–**E**) Comparison of chao, shannon, ace, and sobs indices based on ASV level among different groups. (**F**) PCoA of the fecal microbial communities based on ASV level among different groups. Measurement data were analyzed by Student’s *t*-test and represented as mean ± SEM (n = 6). ** *p* < 0.01 in comparison to the control group; ## *p* < 0.01 in contrast to the model group.

Subsequently, feces from Section 2.5.5 underwent 16S rDNA (V3 + V4) sequencing to assess how sub-chronic HPP2 pre-treatment altered microbial richness and composition. As shown in Figure 8A and Table S1, a Venn disgram was used to display both the shared and unique ASVs among different groups [8,24]. The control, model, SER_15, and HPP2_200 groups exhibited 1452, 925, 1320, and 1480 unique ASVs, respectively. Across all groups, 246 ASVs overlapped; the model group alone lost unique variants relative to controls. In contrast, the SER_15 and HPP2-200 groups had higher numbers of unique ASVs compared to the model group. Figure 8B–E and Table S2 shows all four α-diversity indices (chao, shannon, ace, and sobs) differed between control and model mice on ASV level (*p* < 0.01). Nine-day SER (15 mg/kg) or HPP2 (200 mg/kg) pre-treatment restored the chao, ace, and sobs indices (*p* < 0.01), reversing PTSD-like microbiota depletion. β-diversity (Bray–Curtis PCoA) showed samples from HPP2_200 group clustering with controls, confirming community rescue (Figure 8F).

**Figure 9:**
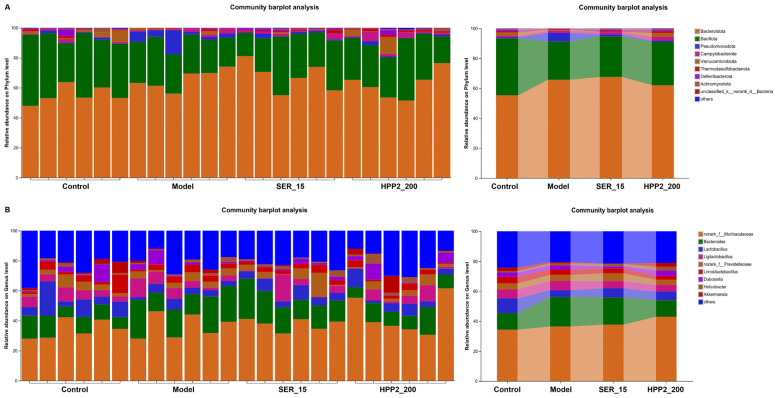
Effects of sub-chronic pre-treatment with HPP2 on the composition of the gut microbiota on phylum and genus levels in foot-shock mice. (**A**) Bacterial composition of the different communities on phylum level among different groups. (**B**) Bacterial composition of the different communities on genus level among different groups. Measurement data were represented as mean ± SEM (n = 6).

**Figure 10:**
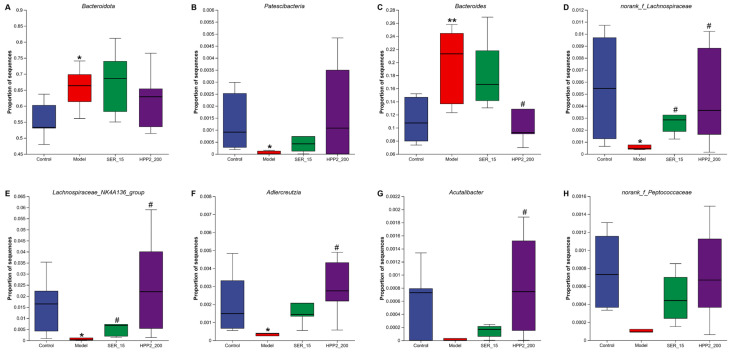
Effects of sub-chronic pre-treatment with HPP2 on the relative abundance of the gut microbiota on phylum and genus levels in foot-shock mice. (**A**,**B**) The relative abundance of *Bacteroidota* and *Patescibacteria* among different groups on phylum level. (**C**–**H**) The relative abundances of *Bacteroides*, *norank_f_Lachnospiraceae*, *Lachnospiraceae_NK4A136_group*, *Adlercreutzia*, *Acutalibacter*, and *norank_f_Peptococcaceae* among different groups on genus level. Measurement data were represented as mean ± SEM (n = 6). * *p* < 0.05, ** *p* < 0.01 relative to the control group; # *p* < 0.05 in comparison to the model group.

We then compared microbial abundance and composition across groups. Heat-maps in Figure 9A,B revealed marked phylum- and genus-level shifts in microbial composition. Phylum-level analysis (Figure 10A,B and Table S3) showed the foot-shock procedure raised *Bacteroidota* and lowered *Patescibacteria* versus control (*p* < 0.05); HPP2 (200 mg/kg) trended toward reversal (*p* > 0.1). Genus-level shifts (Figure 10C–F and Table S4) included disrupted abundances of *Bacteroides*, *norank_f_Lachnospiraceae*, *Lachnospiraceae_NK4A136_group,* and *Adlercreutzia* (*p* < 0.05 or *p* < 0.01); HPP2 (200 mg/kg) normalized their abundance (*p* < 0.05). It was also observed that SER (15 mg/kg) effectively modulated the *norank_f_Lachnospiraceae* and *Lachnospiraceae_NK4A136_group* abundance on genus level (*p* < 0.05). Similarly, the foot-shock mice displayed abnormal abundance of *Acutalibacter* and *norank_f_Peptococcaceae*, and sub-chronic pre-treatment with HPP2 and SER could restore the abundance of these bacteria to some extent. *Acutalibacter* abundance differed between model and HPP2_200 groups (Figure 10G,H; *p* < 0.05). Collectively, the data indicated that sub-chronic HPP2 pre-treatment most effectively offset the foot-shock-induced gut microbiota disruption.

## 4. Discussion

It is widely recognized that *H. perforatum* effectively alleviates depression while exhibiting few side effects [9]. LYT and SG, two typical natural antidepressants derived from *H. perforatum*, hold a front-line position in the clinical treatment of depression [3,25]. As described in the European Pharmacopoeia, LYT is produced from the dried flowering tops of *H. perforatum* using a suitable procedure with ethanol or methanol at a volume fraction of 50–80% [26]. According to the Pharmacopoeia of the People’s Republic of China, SG is composed of the 70% ethanol extract of *H. perforatum* and the water extract of *Acanthopanax senticosus* [27]. Both LYT and SG are made of the alcohol-soluble extract of *H. perforatum*, which primarily contains hypericin, pseudohypericin, hyperforin, hyperoside, and other bioactive ingredients [28]. However, the alcohol-insoluble polysaccharides present in the herb residues generated during the production of LYT and SG, which may hold significant promise for the development of novel therapeutic agents in mental disorders, have yet to be explored.

Our present study is the first to investigate the physicochemical properties of the alcohol-insoluble polysaccharides derived from *H. perforatum*, as well as their therapeutic potential as candidates for the treatment of depression and PTSD. First, HPP1 was extracted using distilled water and precipitated with 70% ethanol, whereas HPP2 was first extracted with 70% ethanol, followed by extraction of the herb residues with distilled water and subsequent precipitation with 70% ethanol. The colorimetric assays, CE, HPGPC, and FT-IR spectroscopy revealed the physicochemical differences between HPP1 and HPP2. Notably, HPP1 exhibits overwhelming glucose content, while HPP2 possesses a more diverse and balanced monosaccharide composition. It is worth exploring whether a high glucose content or a diversified monosaccharide profile exerts a more significant influence on the therapeutic effects of polysaccharides in treating mental disorders.

The FST and TST, two standard behavioral tests utilized for the rapid screening of antidepressant drugs, were initially employed in our study to compare the antidepressant-like effects of acute pre-treatment with HPP1 and HPP2 in mice [29]. Concurrently, the fast-acting antidepressant DLX was selected as the positive control to assess the predictive validity of the animal models [30,31]. The results indicated that acute pre-treatment with DLX (20 mg/kg) significantly reduced the immobility time of mice in both the FST and TST, thereby confirming the robust predictive validity of our behavioral despair models. HPP1 and HPP2 also mitigated the despair behavior of mice in the FST and TST to varying extents, with HPP2 exhibiting superior modulatory effects compared to HPP1. As previously documented, polysaccharides with a high glucose content typically exhibit substantial antidepressant-like effects [5,8]. Accordingly, our study preliminarily revealed that the diverse and balanced monosaccharide profile of HPP2 also plays a significant role in its therapeutic effects on depression.

Immediately thereafter, a comprehensive evaluation of the antidepressant-like effects of HPP2 were conducted in behavioral despair mice. To start with, the FST and TST were employed to systematically assess the effects of acute pre-treatment with HPP2 at doses of 50, 200, and 800 mg/kg, using the first-line TCM antidepressant SG as the positive control [3]. The results indicated that acute pre-treatment with HPP2 one hour prior to the behavioral tests significantly alleviated the behavioral despair of mice exhibited in the stressful situation, and SG also demonstrated positive modulatory effects as previously reported [32]. Subsequently, a 9-day prolonged pre-treatment regimen was implemented, accompanied by the TST, FST, and reserpine-induced ptosis and hypothermia test on days 7, 8, and 9, respectively, to evaluate the effects of sub-chronic pre-treatment with HPP2 on depression-like models. LYT at a dose of 150 mg/kg was selected as the positive control. The findings revealed that sub-chronic pre-treatment with HPP2 effectively restored the behavioral despair exhibited by mice in the FST and TST and antagonized the ptosis degree in reserpine-induced test, with the dose 200 mg/kg proving to be optimal. Regarding the positive drug, LYT partially antagonized the depression-like behaviors of model mice, of which the therapeutic effects at the tested dose were less pronounced compared to that of SG. Worse still, mice in the LYT_150 group exhibited a gradual decline in body weight relative to other groups, indicating the reported gastrointestinal side effects associated with LYT [33]. To summarize, HHP2 at a dose of 200 mg/kg significantly ameliorated the depression-like symptoms potentially through up-regulating the 5-HTergic system in mice, and SG is a relatively effective positive drug than LYT to assess the predictive validity of depression models during the process of new antidepressant development.

Mounting evidence suggests that decreased 5-HT levels in PTSD-like models are linked to heightened impulsivity, aggression, fear, and depressive symptoms, thereby highlighting the pivotal role of the 5-HTergic system in the pathogenesis and therapeutic approaches for PTSD [34,35]. As demonstrated by the reserpine-induced ptosis and hypothermia test, sub-chronic pre-treatment with HPP2 at a dose of 200 mg/kg for 9 days significantly reduced the ptosis degree in reserpine-induced mice by up-regulating the 5-HTergic system. This finding suggested that HPP2 might exert potential anti-PTSD-like effects that were worth exploring. Consequently, a typical PTSD-like model, the foot-shock mice, was established in our study to evaluate the potential anti-PTSD effects of HPP2 [20,36], with SER serving as the positive control drug [37]. Consequently, the foot-shock procedure induced a series of abnormal symptoms, such as a sharp decline in body weight, prolonged freezing time during traumatic re-experiencing, and increased vigilance when facing a new environment. Sub-chronic pre-treatment with SER (15 mg/kg) effectively mitigated the body weight decline in PTSD-like mice, alleviated the fear response to the context, and relieved anxiety-like behaviors in the EPMT and OFT. HPP2 at a dose of 200 mg/kg exerted comparable modulatory effects in the aforementioned behavioral tests. Notably, a 7-day pre-treatment regimen with SER before the foot-shock procedure resulted in a slow body weight gain in normal mice, which was not observed in the HPP2_200 group. This finding was consistent with the reported side effect that patients treated with SER exhibited a significantly decreased appetite [38].

As previously reported, traumatic stresses usually elicit cerebral pathological changes and neurotrophic disturbance in patients and rodents. Studies utilizing magnetic resonance imaging have demonstrated that individuals diagnosed with PTSD exhibited a reduction in hippocampal volume compared to unaffected controls [39]. The negative changes in the number and structure of neuronal cells in the DG, CA1, and CA2/CA3 regions were also observed in PTSD-like models previously [18,40,41]. BDNF, a key molecule involved in neurotrophism and neuroplasticity, has been found to accelerate fear memory extinction and plays important roles in PTSD-like models [42]. In our study, the foot-shock mice displayed an abnormal appearance and decreased neuronal count in the hippocampus, especially in the DG region. A significant reduction in the BDNF expression was also observed in the hippocampal tissues of mice after PTSD modeling. By contrast, HPP2 administration successfully normalized pathological changes and increased BDNF expression in the hippocampus. The results indicated that HPP2 was beneficial to alleviate pathological changes and neurotrophic disturbance in foot-shock mice, which might promote the processes of synapse formation, neuronal maturation, neurogenesis, and synaptic plasticity [43].

Additionally, numerous studies have highlighted the promise of leveraging the gut–brain axis to create innovative therapies for neuropsychiatric conditions [44,45]. As documented in prior research, polysaccharides derived from diverse origins have effectively mitigated neuropsychiatric disorders by modulating the gut microbiota [3,46,47]. Our findings revealed that the unmber of unique ASVs, as well as the chao, ace, and sobs indices on ASV level, were significantly diminished in PTSD-like mice, suggesting the adverse effects of the foot-shock procedure on the richness of the gut microbiota. Pre-treatment with SER (15 mg/kg) or HPP2 (200 mg/kg) successfully restored the above indices to normal levels. Simultaneously, the structure and proportional distribution of the gut microbiota on phylum and genus levels became significantly disrupted in foot-shock mice, and pre-treatment with HPP2 exerted significant modulatory effects on the significant bacteria. As a natural bioactive ingredient, HPP2 might engage in extensive interactions with the imbalanced gut microbiota, thereby generating a diverse array of microbial neuroactive metabolites. These small bioactive molecules could potentially alleviate mild colonic inflammation, repair the intestinal barrier, and thus modulate the disrupted richness and composition of the gut microbiota.

Future studies could explore multiple directions to further evaluate HPP2’s therapeutic potential for depression and PTSD. First, it is necessary to isolate the individual polysaccharide-rich sub-fractions that make up HPP2 and determine their monosaccharide sequence, linkage pattern, anomeric configuration, degree of methyl-esterification/acetylation and molecular weight. Second, to validate the causal role of the gut–brain axis, we will recolonise germ-free or ABX-treated mice with HPP2-conditioned microbiota and test whether the behavioural phenotype is transferable. Third, we would like to expand the stress model repertoire (chronic social defeat, predator scent, and single-prolonged stress) to ensure that efficacy is not paradigm-dependent. Last but not least, a 28-day GLP repeat-dose tox study in two species (rodent and non-rodent) with full clinical pathology, histopathology, and cardiovascular telemetry is essential in the future study.

## 5. Conclusions

The present study provided two novel polysaccharide-rich ingredients from *H. perforatum*, HPP1 and HPP2, of which the physicochemical characteristics were elucidated across the board. Comparatively, HPP2 exerted significant modulatory effects in depression-like and PTSD-like mouse models. The effects might be mediated through restoring hippocampal pathological changes and neurotrophic disturbance, as well as balancing the dysregulated gut microbiota. To our knowledge, this study is the first to investigate the alcohol-insoluble polysaccharides from *H. perforatum*, especially their potential therapeutic effects on neuropsychiatric disorders. Although further studies are needed to confirm the molecular targets and mechanisms of HPP2 and to identify who is unlikely to benefit from HPP2 therapy, our research has uncovered a promising therapeutic candidate for depression and PTSD that warrants additional investigation and exploration.

## Figures and Tables

**Table 1:** Content and monosaccharide composition analyses of HPP1 and HPP2.

Sample	Content (*w*/*w*, %)		Monosaccharide Composition (Ratio)				
	Neutral Sugar	Uronic Acid	Arabinose	Glucose	Galactose	Mannose	Galacturonic Acid
HPP1	44.3	58.6	1.00	9.03	2.44	0.75	2.32
HPP2	35.6	57.4	1.00	1.42	1.41	0.19	0.57

**Table 2:** Effects of sub-chronic pre-treatment with HPP2 on the 5-HTergic and NEergic systems by reserpine-induced ptosis and hypothermia test in mice.

Group	Score of Ptosis	ΔT/°C
Control	3.4 ± 0.2	5.7 ± 0.6
LYT_150	2.6 ± 0.2 *	4.2 ± 0.3
HPP2_50	2.8 ± 0.3	5.0 ± 0.2
HPP2_200	2.1 ± 0.2 ***	4.8 ± 0.3
HPP2_800	2.6 ± 0.2 *	5.7 ± 0.2

LYT (150 mg/kg, i.g.) and HPP2 (50, 200, and 800 mg/kg, i.g.) were pre-treated as described in Section 2.4.2. Data were analyzed by Student’s *t*-test or one-way ANOVA (post hoc LSD test), and presented as mean ± SEM (n = 10). * *p* < 0.05, *** *p* < 0.001 compared to the control group.

## Data Availability

Data utilized for supporting research discoveries remain obtainable from corresponding author as required.

## Supplementary Materials

The following supporting information can be downloaded at: https://www.mdpi.com/article/10.3390/nu17203222/s1, Table S1: Sequences for each sample on ASV level; Table S2: α diversity estimators for each sample on ASV level; Table S3: Relative abundance of gut microbiota among different groups on phylum level; Table S4: Relative abundance of gut microbiota among different groups on genus level.

## Abbreviations

The following abbreviations are used in this manuscript:

ANOVA	Analysis of variance
ASV	Amplicon sequence variant
BDNF	Brain-derived neurotrophic factor
CA	Cornu ammonis
CE	Capillary electrophoresis
CFT	Contextual freezing test
DAPI	4′,6-Diamidino-2-phenylindole
DG	Dentate gyrus
DLX	Duloxetine hydrochloride
EPMT	Elevated plus maze test
FST	Forced swimming test
FT-IR	Fourier transform infrared
*H. perforatum*	*Hypericum perforatum* L.
HE	Hematoxylin & eosin
HPGPC	High-performance gel permeation chromatography
HPP	Polysaccharides from *H. perforatum*
IF	Immunofluorescence
LYT	*Luyoutai*
MW	Molecular weight
NE	Noradrenaline
OFT	Open field test
PCoA	Principal coordinate analysis
PTSD	Post-traumatic stress disorder
SEM	Standard error media
SER	Sertraline hydrochloride
SG	*Shuganjieyu* capsule
TST	Tail suspension test
5-HT	Serotonin
