# Supplementary material for: A Polysaccharide-Rich Ingredient from Hypericum perforatum L. Ameliorates Depression-like and Post-Traumatic Stress Disorder-like Symptoms in Mouse Models
Source: Nutrients. 2025 Oct 14;17(20):3222. doi: 10.3390/nu17203222 (PMC12567441; doi:10.3390/nu17203222)
Supplement: Supplementary file 1 [file nutrients-17-03222-s001.zip › nutrients-3897169-supplementary.pdf]

A Polysaccharide-Rich Ingredient from *Hypericum perforatum* L. Ameliorates Depression-Like and Post-Traumatic Stress Disorder-Like Symptoms in Mouse Models

Zi-Jia Jin [1,2,†], Shuai-Ming Zhu [2,†], Fu-Yao Luo [2,†], Yue Sun [2,3], Chun-Xue Gao [2,3], Ting Feng [2,4], Hao Ma [2], Rui Xue [2], Chang-Wei Li [2], Lei An [1,*], You-Zhi Zhang [2,*]

1 Key Laboratory of Geriatric Nutrition and Health, Ministry of Education, Beijing Technology and Business University, Beijing 100048, China

2 Academy of Military Medical Sciences, Beijing 100850, China

3 School of Pharmacy, North China University of Science and Technology, Tangshan 063210, China

4 Nanjing University of Chinese Medicine, Nanjing 210046, China

* Correspondence: anlei@btbu.edu.cn (L.A.); bcczyz@163.com (Y.-Z.Z.)

† These authors share first authorship.

Contents ························· Page

1) **Table S1.** Sequences for each sample on ASV level ··· 2
2) **Table S2.** α diversity estimators for each sample on ASV level ··· 3
3) **Table S3.** Relative abundance of gut microbiota among different groups on phylum level ··· 4
4) **Table S4.** Relative abundance of gut microbiota among different groups on genus level ··· 5

Table S1. Sequences for each sample on ASV level

Sample name	OTU number	Sequence
Control-1	374	34135
Control-2	649	34135
Control-3	514	34135
Control-4	428	34135
Control-5	517	34135
Control-6	469	34135
Model-1	276	34135
Model-2	392	34135
Model-3	321	34135
Model-4	265	34135
Model-5	358	34135
Model-6	307	34135
SER_15-1	433	34135
SER_15-2	424	34135
SER_15-3	377	34135
SER_15-4	475	34135
SER_15-5	475	34135
SER_15-6	554	34135
HPP2_200-1	533	34135
HPP2_200-2	487	34135
HPP2_200-3	342	34135
HPP2_200-4	564	34135
HPP2_200-5	610	34135
HPP2_200-6	405	34135

A total of 5811 ASVs were screened from all four groups.

Table S2. α diversity estimators for each sample on ASV level

Sample name	Chao	Shannon	Ace	Sobs
Control-1	375	4.17	379	374
Control-2	743	4.37	762	649
Control-3	548	3.97	566	514
Control-4	434	4.19	439	428
Control-5	527	4.36	538	517
Control-6	479	4.32	489	469
Model-1	279	3.80	283	276
Model-2	397	3.81	404	392
Model-3	321	4.07	321	321
Model-4	266	3.62	269	265
Model-5	359	3.78	363	358
Model-6	310	3.93	315	307
SER_15-1	436	3.95	441	433
SER_15-2	427	3.93	432	424
SER_15-3	379	3.90	384	377
SER_15-4	475	4.08	478	475
SER_15-5	479	4.07	484	475
SER_15-6	606	3.83	623	554
HPP2_200-1	543	4.14	554	533
HPP2_200-2	489	4.15	495	487
HPP2_200-3	342	3.65	344	342
HPP2_200-4	568	4.62	576	564
HPP2_200-5	620	4.70	630	610
HPP2_200-6	408	4.02	413	405

Student's t-test was used for statistical analyses of above estimators between two groups.

Table S3. Relative abundance of gut microbiota among different groups on phylum level

Species Name	Control		Model		SER_15		HPP2_200	
	Mean(%)	SD(%)	Mean(%)	SD(%)	Mean(%)	SD(%)	Mean(%)	SD(%)
p_Bacteroidota	55.2700	5.6750	65.6800	6.6220	67.6000	9.8000	62.0900	9.1440
p_Bacillota	38.0700	8.0980	25.7200	4.5880	27.1800	8.5310	28.9000	7.6840
p_Pseudomonadota	0.7427	0.7117	5.5930	5.4060	1.0980	1.0160	0.8703	0.9349
p_Campylobacterota	0.9245	0.7132	1.3500	0.9552	1.6410	1.3220	2.5010	2.1550
p_Verrucomicrobiota	2.2980	2.7890	0.7857	0.9658	0.1814	0.3566	2.6220	4.5120
p_Thermodesulfobacteriota	0.6044	0.5662	0.3880	0.4626	0.7138	0.6218	1.1220	0.3766
p_Deferribacterota	0.9276	1.6850	0.2705	0.2524	0.7011	0.4142	0.7173	1.2690
p_Actinomycetota	0.7989	0.9446	0.0623	0.0525	0.4868	0.3268	0.6342	0.6403
p_unclassified_k_norank_d_Bacteria	0.1363	0.0467	0.1185	0.0347	0.1545	0.0763	0.1763	0.0515
p_Cyanobacteriota	0.1008	0.1805	0.0218	0.0289	0.1788	0.1009	0.1915	0.2533
p_Patescibacteria	0.1297	0.1207	0.0046	0.0071	0.0633	0.0771	0.1748	0.1985
p_Chloroflexota	0.0000	0.0000	0.0000	0.0000	0.0010	0.0025	0.0000	0.0000

Student's t-test was used for statistical analyses of above estimators between two groups.

Table S4. Relative abundance of gut microbiota among different groups on genus level

Species Name	Control		Model		SER_15		HPP2_200	
	Mean(%)	SD(%)	Mean(%)	SD(%)	Mean(%)	SD(%)	Mean(%)	SD(%)
g_norank_f_Muribaculaceae	34.3200	6.0310	36.4200	7.9220	37.6500	3.8300	1.7667	0.6222
g_Bacteroides	11.1200	3.2900	19.8000	5.8180	18.1900	5.2650	10.9133	0.0122
g_Lactobacillus	9.7760	7.0650	4.4400	1.9090	6.1450	2.2880	3.3133	0.3458
g_Ligilactobacillus	5.8710	2.1410	6.2090	3.8620	4.6900	6.7630	2.5800	0.4610
g_norank_f_Prevotellaceae	4.1030	2.6120	3.9810	1.6250	5.2360	5.7610	0.3800	0.9443
g_Limosilactobacillus	4.4550	4.1970	3.6090	1.2930	3.2520	1.1250	1.1400	0.7674
g_Dubosiella	2.8950	4.5690	2.7660	3.5640	1.4360	1.1230	0.1600	0.9838
g_Helicobacter	0.9245	0.7132	1.3500	0.9552	1.6410	1.3220	4.3333	0.2276
g_Akkermansia	2.2980	2.7890	0.7857	0.9658	0.1814	0.3566	6.0982	0.1069
g_unclassified_f_Lachnospiraceae	1.6360	1.3260	1.1050	2.3580	1.3720	1.2630	5.0239	0.1701
g_Escherichia-Shigella	0.2406	0.5850	4.3850	5.6210	0.4453	0.5999	10.9535	0.0120
g_unclassified_f_Prevotellaceae	0.4357	0.2704	2.2730	2.0500	1.6740	1.1620	6.2267	0.1011
g_Lachnospiraceae_NK4A136_group	1.5920	1.2560	0.1292	0.2290	0.6803	0.5562	10.8867	0.0124
g_norank_o_Clostridia_UCG-014	1.2790	1.1310	0.4645	0.2494	1.2030	0.3357	6.7467	0.0804
g_Parabacteroides	0.8870	0.3608	1.1130	0.7751	1.5360	0.8400	3.0563	0.3830
g_Prevotellaceae_UCG-001	0.7386	0.3964	0.4985	0.1506	1.1470	0.5542	3.8467	0.2785
g_Thomasclavelia	0.6636	1.2280	2.0470	2.1560	0.5456	0.3735	4.6303	0.2010
g_Rikenellaceae_RC9_gut_group	1.4930	0.6061	0.5228	0.5776	0.5557	0.3180	6.9580	0.0733
g_Turicimonas	0.4590	0.5054	1.1780	0.6085	0.6378	0.4423	6.5295	0.0885

g_Ruminococcus	0.9691	1.2420	0.6707	1.0730	0.3749	0.4697	2.9542	0.3987
g_Blautia	0.8212	1.7030	0.1434	0.1723	1.5960	1.7150	6.6223	0.0850
g_Anaerotruncus	1.0800	0.8258	0.3698	0.1840	0.5517	0.1688	4.0200	0.2593
g_Mucispirillum	0.9276	1.6850	0.2705	0.2524	0.7011	0.4142	3.9967	0.2618
g_norank_f_Desulfovibrionaceae	0.3987	0.4705	0.3754	0.4449	0.6793	0.6114	8.7800	0.0324
g_unclassified_f_Ruminococcaceae	0.4240	0.4605	0.7984	1.3000	0.6884	0.4049	3.0200	0.3886
g_Candidatus_Arthromitus	0.9858	0.6401	0.4240	0.1580	0.5350	0.1712	7.2263	0.0650
g_Alistipes	0.6672	0.3774	0.6008	0.5389	0.5046	0.2371	0.4919	0.9207
g_norank_f_Lachnospiraceae	0.5547	0.4159	0.0800	0.0769	0.3531	0.2838	8.2903	0.0404
g_Rikenella	0.3921	0.2884	0.0633	0.0781	0.3886	0.2715	8.0854	0.0443
g_[Eubacterium]_ventriosum_group	0.4048	0.6611	0.0927	0.0966	0.4301	0.8435	0.0853	0.9935
g_Muribaculum	0.2806	0.0876	0.2442	0.1751	0.2345	0.0706	1.2289	0.7461
g_Colidextribacter	0.1935	0.1454	0.1824	0.0769	0.2958	0.2364	2.3120	0.5102
g_Bifidobacterium	0.4990	0.9763	0.0238	0.0474	0.1530	0.2206	3.2019	0.3615
g_norank_f_[Eubacterium]_coprostanoligenes_group	0.3723	0.2897	0.0730	0.0555	0.1120	0.1083	8.0854	0.0443
g_Longibaculum	0.1327	0.1047	0.5081	0.6634	0.0897	0.0770	1.4923	0.6840
g_Odoribacter	0.5218	0.7351	0.0086	0.0101	0.2026	0.2148	7.8635	0.0489
g_[Ruminococcus]_gnavus_group	0.7092	1.6450	0.1170	0.2146	0.0000	0.0000	6.1598	0.1041
g_Adlercreutzia	0.2057	0.1684	0.0360	0.0247	0.2335	0.2420	12.1701	0.0068
g_Mediterraneibacter	0.1763	0.3832	0.0101	0.0220	0.4671	0.7443	7.6769	0.0532
g_Massiliomicrobiota	0.0562	0.0336	0.2143	0.3070	0.2092	0.3054	1.4151	0.7020
g_unclassified_o_Bacteroidales	0.1282	0.0313	0.1535	0.0573	0.1687	0.0759	2.6151	0.4549
g_unclassified_k_norank_d	0.1363	0.0467	0.1185	0.0347	0.1545	0.0763	2.9229	0.4037

_Bacteria								
g_unclassified_f_Erysipelatoclostridiaceae	0.2280	0.5259	0.1236	0.2939	0.2001	0.3726	2.8988	0.4075
g_norank_f_Erysipelotrichaceae	0.0963	0.1147	0.1763	0.1063	0.0887	0.0581	3.7916	0.2849
g_A2	0.3794	0.8848	0.0096	0.0236	0.0578	0.1415	5.6894	0.1277
g_norank_o_RF39	0.0912	0.1091	0.0861	0.0864	0.0927	0.0878	5.2091	0.1571
g_Roseburia	0.2467	0.3030	0.0279	0.0318	0.0578	0.0394	4.9765	0.1735
g_Clostridium	0.2173	0.3367	0.1702	0.3656	0.0000	0.0000	8.6171	0.0348
g_Candidatus_Saccharimonas	0.1297	0.1207	0.0046	0.0071	0.0633	0.0771	8.4839	0.0370
g_Zag_111	0.0745	0.1589	0.0137	0.0293	0.1104	0.0669	7.0027	0.0718
g_Prevotellaceae_NK3B31_group	0.1383	0.1479	0.0056	0.0099	0.0846	0.0825	7.1317	0.0678
g_Marvinbryantia	0.0466	0.0714	0.0699	0.0705	0.1900	0.2839	4.6973	0.1954
g_norank_o_Clostridia_vadinBB60_group	0.1221	0.0965	0.0101	0.0153	0.0633	0.0611	7.0098	0.0716
g_norank_f_Anaerovoracaceae	0.0466	0.0472	0.0988	0.0824	0.1135	0.1108	3.5224	0.3179
g_Christensenellaceae_R-7_group	0.0547	0.0471	0.0446	0.0366	0.0973	0.0626	2.7677	0.4288
g_Desulfovibrio	0.1930	0.1754	0.0086	0.0103	0.0269	0.0151	9.9141	0.0193
g_Lachnospiraceae_UCG-006	0.0218	0.0171	0.0000	0.0000	0.0096	0.0106	10.1232	0.0176
g_Allobaculum	0.2503	0.4384	0.0000	0.0000	0.0000	0.0000	9.8053	0.0203
g_unclassified_f_Lactobacillaceae	0.0572	0.0364	0.0588	0.0522	0.0633	0.0429	0.2431	0.9703
g_Negativibacillus	0.0537	0.0676	0.0750	0.0582	0.0542	0.0413	0.8948	0.8267
g_unclassified_f_Oscillospiraceae	0.0461	0.0539	0.0157	0.0299	0.0892	0.1036	6.3046	0.0977

g_norank_f_Peptococcaceae	0.0770	0.0406	0.0117	0.0101	0.0471	0.0266	10.7594	0.0131
g_Extibacter	0.0000	0.0000	0.0167	0.0353	0.1586	0.3884	2.3119	0.5103
g_Faecalibaculum	0.0385	0.0622	0.0380	0.0551	0.0309	0.0182	0.6095	0.8943
g_Acutalibacter	0.0598	0.0516	0.0086	0.0196	0.0142	0.0094	6.2469	0.1002
g_[Eubacterium]_xylanophilum_group	0.0897	0.0945	0.0081	0.0158	0.0233	0.0381	5.3419	0.1484
g_unclassified_f_Eggerthellaceae	0.0537	0.0555	0.0015	0.0025	0.0522	0.0442	9.6689	0.0216
g_[Eubacterium]_siraeum_group	0.0137	0.0201	0.0101	0.0195	0.0233	0.0258	5.0710	0.1667
g_Paludicola	0.0334	0.0498	0.0187	0.0227	0.0831	0.0350	8.4189	0.0381
g_Anaeroplasma	0.0648	0.0451	0.0142	0.0249	0.0142	0.0081	4.9747	0.1737
g_[Ruminococcus]_gauvreauii_group	0.0679	0.1505	0.0117	0.0285	0.0583	0.0963	2.7891	0.4253
g_norank_f_Oscillospiraceae	0.0289	0.0429	0.0076	0.0083	0.0253	0.0283	2.4900	0.4771
g_CAG-196	0.0253	0.0278	0.0046	0.0054	0.0664	0.0394	7.0518	0.0703
g_unclassified_p_Bacillota	0.0253	0.0221	0.0127	0.0161	0.0370	0.0409	3.9427	0.2677
g_GCA-900066575	0.0167	0.0248	0.0046	0.0076	0.0491	0.0821	5.5564	0.1353
g_Monoglobus	0.0339	0.0235	0.0213	0.0330	0.0208	0.0111	2.4310	0.4879
g_Lachnoclostridium	0.0517	0.0495	0.0020	0.0050	0.0182	0.0323	7.1866	0.0662
g_norank_o_Rhodospirillales	0.0324	0.0328	0.0162	0.0089	0.0117	0.0080	3.3351	0.3428
g_Streptococcus	0.0613	0.0695	0.0015	0.0037	0.0091	0.0195	5.5484	0.1358
g_NK4A214_group	0.0228	0.0221	0.0025	0.0062	0.0223	0.0176	9.4526	0.0238
g_norank_f_UCG-010	0.0243	0.0259	0.0035	0.0087	0.0269	0.0284	6.3665	0.0951
g_unclassified_c_Bacilli	0.0248	0.0258	0.0269	0.0254	0.0167	0.0294	2.2976	0.5130
g_Oscillibacter	0.0258	0.0400	0.0020	0.0031	0.0213	0.0325	3.4384	0.3288
g_Enterocloster	0.0623	0.0965	0.0000	0.0000	0.0046	0.0085	2.7362	0.4341

g_Coriobacteriaceae_UCG-002	0.0193	0.0218	0.0000	0.0000	0.0213	0.0273	5.9878	0.1122
g_norank_f_Ruminococcaceae	0.0263	0.0238	0.0020	0.0050	0.0243	0.0271	8.6383	0.0345
g_norank_f_Flavobacteriaceae	0.0350	0.0380	0.0010	0.0025	0.0076	0.0074	6.2301	0.1009
g_unclassified_c_Clostridia	0.0132	0.0150	0.0086	0.0083	0.0228	0.0177	2.6558	0.4478
g_Harryflintia	0.0117	0.0244	0.0000	0.0000	0.0345	0.0462	10.3061	0.0161
g_Agathobaculum	0.0309	0.0302	0.0000	0.0000	0.0101	0.0089	10.4197	0.0153
g_Erysipelotrichaceae_UCG-003	0.0000	0.0000	0.0000	0.0000	0.0557	0.1182	6.2609	0.0996
g_Intestinimonas	0.0152	0.0263	0.0035	0.0062	0.0066	0.0103	3.3257	0.3441
g_Ruthenibacterium	0.0152	0.0214	0.0132	0.0323	0.0177	0.0203	2.8442	0.4163
g_Gordonibacter	0.0167	0.0173	0.0000	0.0000	0.0177	0.0104	9.3394	0.0251
g_[Eubacterium]_brachy_group	0.0056	0.0068	0.0030	0.0038	0.0127	0.0103	7.2230	0.0651
g_Anaerofustis	0.0015	0.0037	0.0258	0.0398	0.0000	0.0000	4.7845	0.1883
g_unclassified_f_Erysipelotrichaceae	0.0081	0.0103	0.0041	0.0099	0.0111	0.0244	2.6538	0.4481
g_Candidatus_Soleaferrea	0.0025	0.0040	0.0015	0.0037	0.0081	0.0094	5.1396	0.1619
g_Anaerostipes	0.0051	0.0124	0.0000	0.0000	0.0056	0.0099	1.9483	0.5832
g_norank_f_Erysipelatoclostridiaceae	0.0020	0.0050	0.0000	0.0000	0.0000	0.0000	2.0942	0.5531
g_Bilophila	0.0117	0.0220	0.0041	0.0099	0.0076	0.0136	0.6199	0.8919
g_ASF356	0.0132	0.0164	0.0041	0.0063	0.0046	0.0098	1.3286	0.7223
g_UCG-005	0.0030	0.0074	0.0010	0.0025	0.0096	0.0103	4.6830	0.1965
g_UCG-003	0.0106	0.0219	0.0000	0.0000	0.0000	0.0000	6.4241	0.0927
g_Family_XIII_AD3011_group	0.0035	0.0062	0.0020	0.0050	0.0015	0.0037	3.4200	0.3313

g_Anaerotignum	0.0000	0.0000	0.0041	0.0085	0.0081	0.0184	3.6260	0.3048
g_Butyribacter	0.0051	0.0110	0.0000	0.0000	0.0117	0.0231	2.5104	0.4734
g_Defluviitaleaceae_UCG-011	0.0086	0.0142	0.0000	0.0000	0.0010	0.0025	4.3694	0.2242
g_Family_XIII_UCG-001	0.0081	0.0130	0.0000	0.0000	0.0046	0.0112	4.5829	0.2050
g_Faecalimonas	0.0056	0.0137	0.0000	0.0000	0.0000	0.0000	6.3019	0.0978
g_unclassified_f_Muribaculaceae	0.0041	0.0037	0.0000	0.0000	0.0041	0.0057	5.8599	0.1186
g_unclassified_o_Oscillospirales	0.0025	0.0062	0.0000	0.0000	0.0051	0.0046	5.9786	0.1127
g_unclassified_f_Sutterellaceae	0.0025	0.0040	0.0005	0.0012	0.0000	0.0000	2.3119	0.5103
g_Peptococcus	0.0056	0.0099	0.0000	0.0000	0.0051	0.0087	4.5612	0.2069
g_unclassified_o_Enterobacterales	0.0000	0.0000	0.0076	0.0127	0.0010	0.0025	4.2570	0.2350
g_HT002	0.0066	0.0161	0.0000	0.0000	0.0000	0.0000	2.0942	0.5531
g_norank_f_Eggerthellaceae	0.0020	0.0050	0.0010	0.0025	0.0035	0.0087	1.1096	0.7747
g_UCG-007	0.0015	0.0025	0.0000	0.0000	0.0030	0.0074	1.9483	0.5832
g_Butyricimonas	0.0010	0.0025	0.0015	0.0037	0.0030	0.0051	2.3854	0.4963
g_unclassified_f_Gastranaerophilaceae	0.0005	0.0012	0.0035	0.0087	0.0005	0.0012	0.0356	0.9982
g_Anaerovorax	0.0020	0.0037	0.0000	0.0000	0.0000	0.0000	4.5684	0.2063
g_unclassified_f_Enterobacteriaceae	0.0000	0.0000	0.0030	0.0074	0.0020	0.0050	2.0942	0.5531
g_UCG-009	0.0041	0.0074	0.0000	0.0000	0.0010	0.0025	4.1263	0.2481
g_norank_f_Sutterellaceae	0.0051	0.0097	0.0000	0.0000	0.0000	0.0000	6.2609	0.0996
g_norank_f_Christensenellaceae	0.0015	0.0037	0.0015	0.0037	0.0000	0.0000	1.9463	0.5836
g_Tyzzerella	0.0046	0.0098	0.0000	0.0000	0.0000	0.0000	6.2609	0.0996

g_Paraprevotella	0.0000	0.0000	0.0015	0.0037	0.0025	0.0062	2.0942	0.5531
g_unclassified_f_Desulfovibrionaceae	0.0010	0.0025	0.0000	0.0000	0.0000	0.0000	6.8359	0.0773
g_Enterococcus	0.0000	0.0000	0.0015	0.0037	0.0000	0.0000	2.0942	0.5531
g_unclassified_f_Rikenellaceae	0.0020	0.0037	0.0000	0.0000	0.0000	0.0000	3.8838	0.2743
g_Lachnospiraceae_NC2004_group	0.0035	0.0087	0.0000	0.0000	0.0000	0.0000	3.0000	0.3916
g_Acetatifactor	0.0000	0.0000	0.0000	0.0000	0.0000	0.0000	3.0000	0.3916
g_unclassified_c_Gammaproteobacteria	0.0005	0.0012	0.0025	0.0062	0.0000	0.0000	2.0942	0.5531
g_Hydrogenoanaerobacterium	0.0000	0.0000	0.0010	0.0025	0.0015	0.0037	2.0942	0.5531
g_Lachnospiraceae_UCG-008	0.0000	0.0000	0.0000	0.0000	0.0025	0.0062	3.0000	0.3916
g_norank_o_Izemoplasmatales	0.0025	0.0049	0.0000	0.0000	0.0000	0.0000	6.2609	0.0996
g_Eisenbergiella	0.0000	0.0000	0.0000	0.0000	0.0000	0.0000	6.2609	0.0996
g_norank_c_Clostridia	0.0000	0.0000	0.0000	0.0000	0.0025	0.0040	6.2609	0.0996
g_unclassified_o_Erysipelotrichales	0.0005	0.0012	0.0000	0.0000	0.0020	0.0050	2.0942	0.5531
g_unclassified_c_Bacteroidia	0.0000	0.0000	0.0005	0.0012	0.0000	0.0000	4.1263	0.2481
g_unclassified_f_Butyricicoccaceae	0.0015	0.0037	0.0005	0.0012	0.0000	0.0000	2.0942	0.5531
g_[Clostridium]_methylpentosum_group	0.0000	0.0000	0.0000	0.0000	0.0000	0.0000	3.0000	0.3916
g_Lachnospiraceae_UCG-004	0.0020	0.0050	0.0000	0.0000	0.0000	0.0000	3.0000	0.3916
g_Liquorilactobacillus	0.0000	0.0000	0.0000	0.0000	0.0020	0.0050	3.0000	0.3916

g_Lachnospiraceae_FCS020_group	0.0000	0.0000	0.0000	0.0000	0.0000	0.0000	3.0000	0.3916
g_unclassified_f_Atopobiaceae	0.0010	0.0025	0.0000	0.0000	0.0010	0.0025	2.0909	0.5538
g_Solirubrobacter	0.0000	0.0000	0.0000	0.0000	0.0015	0.0037	3.0000	0.3916
g_Berryella	0.0000	0.0000	0.0000	0.0000	0.0015	0.0037	3.0000	0.3916
g_Arthrobacter	0.0000	0.0000	0.0000	0.0000	0.0015	0.0037	3.0000	0.3916
g_norank_o_Oscillospirales	0.0000	0.0000	0.0000	0.0000	0.0000	0.0000	3.0000	0.3916
g_unclassified_o_Lactobacillales	0.0015	0.0025	0.0000	0.0000	0.0000	0.0000	6.2609	0.0996
g_unclassified_o_Coriobacteriales	0.0015	0.0037	0.0000	0.0000	0.0000	0.0000	3.0000	0.3916
g_norank_f_Gastranaerophilaceae	0.0000	0.0000	0.0000	0.0000	0.0015	0.0037	3.0000	0.3916
g_Lachnospiraceae_UCG-010	0.0010	0.0025	0.0000	0.0000	0.0000	0.0000	2.0942	0.5531
g_Caldicoprobacter	0.0000	0.0000	0.0000	0.0000	0.0005	0.0012	2.0942	0.5531
g_norank_f_Mitochondria	0.0005	0.0012	0.0000	0.0000	0.0000	0.0000	2.0942	0.5531
g_unclassified_f_Helicobacteraceae	0.0000	0.0000	0.0000	0.0000	0.0000	0.0000	3.0000	0.3916
g_Brucella	0.0010	0.0025	0.0000	0.0000	0.0000	0.0000	3.0000	0.3916
g_Serratia	0.0010	0.0025	0.0000	0.0000	0.0000	0.0000	3.0000	0.3916
g_norank_o_RBG-13-54-9	0.0000	0.0000	0.0000	0.0000	0.0010	0.0025	3.0000	0.3916
g_unclassified_f_Peptococcaceae	0.0010	0.0025	0.0000	0.0000	0.0000	0.0000	3.0000	0.3916
g_Lachnospiraceae_NK4B4_group	0.0000	0.0000	0.0005	0.0012	0.0000	0.0000	3.0000	0.3916
g_Olsenella	0.0000	0.0000	0.0000	0.0000	0.0000	0.0000	3.0000	0.3916
g_unclassified_f_Anaerovo	0.0000	0.0000	0.0005	0.0012	0.0000	0.0000	3.0000	0.3916

racaceae								
g_unclassified_f_Tannerellaceae	0.0000	0.0000	0.0000	0.0000	0.0005	0.0012	3.0000	0.3916
g_norank_o_Chloroplast	0.0005	0.0012	0.0000	0.0000	0.0000	0.0000	3.0000	0.3916
g_Oscillospira	0.0005	0.0012	0.0000	0.0000	0.0000	0.0000	3.0000	0.3916
g_Aerococcus	0.0005	0.0012	0.0000	0.0000	0.0000	0.0000	3.0000	0.3916
g_Carnobacterium	0.0005	0.0012	0.0000	0.0000	0.0000	0.0000	3.0000	0.3916

Student's t-test was used for statistical analyses of above estimators between two groups.
